# Neutralizing antibody titres to SARS-CoV-2 Omicron variant and wild-type virus in those with past infection or vaccinated or boosted with mRNA BNT162b2 or inactivated CoronaVac vaccines

**DOI:** 10.21203/rs.3.rs-1207071/v1

**Published:** 2022-01-05

**Authors:** Malik Peiris, Samuel Cheng, Chris Ka Pun Mok, Yonna Leung, Susanna Ng, Karl Chan, Fanny Ko, Karen Yiu, Bosco Lam, Eric Lau, Ken Chan, Leo Luk, John Li, Leo Tsang, Leo Poon, Chunke Chen, David Hui

**Affiliations:** University of Hong Kong; University of Hong Kong; Chinese University of Hong Kong; University of Hong Kong; Chinese University of Hong Kong; University of Hong Kong; Chinese University of Hong Kong; Chinese University of Hong Kong; North Lantau Hospital; University of Hong Kong; Chinese University of Hong Kong; University of Hong Kong; University of Hong Kong; University of Hong Kong; University of Hong Kong; Chinese University of Hong Kong; The Chinese University of Hong Kong

**Keywords:** SARS-CoV-2, immunity, protection, vaccine, Omicron, BNT162b2, CoronaVac, SinoVac

## Abstract

Omicron, a novel SARS-CoV-2 variant has emerged and is rapidly becoming the dominant SARS-CoV-2 virus circulating globally. It is important to define reductions in virus neutralizing activity in serum of convalescent or vaccinated individuals to understand potential loss of protection from infection or re-infection. Two doses of BNT162b2 or CoronaVac vaccines provided little 50% plaque reduction neutralization test (PRNT_50_) antibody immunity against the Omicron variant, even at one-month post vaccination. Booster doses with BNT162b2 in those with two doses of either BNT162b2 or CoronaVac provided acceptable neutralizing immunity against Omicron variant at 1-month post-booster dose. However, three doses of BNT162b2 elicited higher levels of PRNT_50_ antibody to Omicron variant suggesting longer duration of protection. Convalescent from SARS-CoV-2 infection did not have protective PRNT_50_ antibody levels to Omicron, but a single dose of BNT162b2 vaccine provided protective immunity. Field vaccine-efficacy studies against Omicron variant against different vaccines are urgently needed.

## Text

A novel SARS-CoV-2 variant with increased transmissibility was first reported in South Africa in November 2021^1^, classified as a variant of concern and named Omicron^2^. The virus had 37 amino acid substitutions in the spike protein of the virus, 15 of them being in the receptor binding domain. It was predicted that some of these amino acid substitutions would have major impact in evading neutralizing antibody. Virus neutralizing antibody is a major determinant of protection from infection in humans and in macaques experimentally challenged with virus^3,4^. Neutralizing antibody thresholds associated with protection from re-infection or severe disease have been reported^5,6^. In experimentally infected macaques convalescent from SARS-CoV-2 infection, depletion of CD8 T cells reduced the protective effect from re-infection, suggesting that T cell immunity can contribute to protection^4^. However, quantitative correlates of T cell protection remain elusive.

Studies on the immunogenicity and vaccine efficacy of BNT162b2 vaccine have been reported, but whole virus inactivated vaccines such as CoronaVac (Sinovac) are less studied. CoronaVac is one of the WHO approved vaccines and over 750 million doses have been administered in more than 40 countries. Phase-three randomized clinical trials of CoronaVac showed efficacies ranging from 50.7% and higher protection from severe disease^7,8^. But there have been reports of breakthrough infections leading to severe disease and death in CoronaVac vaccinated adults^9^. Data on the immunogenicity of current COVID-19 vaccines against Omicron variant, is urgently needed.

We had previously demonstrated that those vaccinated with BNT162b2 had markedly higher levels of geometric mean PRNT_50_ antibody titres compared to those vaccinated with CoronaVac vaccines, at one-month post-second dose of vaccine^10^. Allowing for antibody waning, we estimated that only 16% of the CoronaVac vaccinated individuals would retain protective thresholds against the wild-type virus while 79.6% of BNT162b2 vaccinees would be similarly protected by six months after second dose of vaccine^10^. Subsequently, we randomized the cohort receiving CoronaVac vaccine to receive booster doses of CoronaVac or BNT162b2 and showed marked increase in neutralizing antibody following boosting with BNT162b2 but a less optimal responses with CoronaVac^11^.

However, the emergence of virus variants that are able to partially evade vaccine immunity would compromise vaccine protection. It is important that we rapidly compare virus neutralizing antibody levels to the wild-type virus to which vaccines are developed and Omicron variant, in persons who have received vaccinations or booster doses, for formulation of appropriate public health policy. Here we compare PRNT antibody titres to wild-type SARS-CoV-2 and Omicron variant in previously studied cohorts who recovered from COVID-19^12^, uninfected community subjects one month after receiving two doses of BNT162b2 or CoronaVac vaccine^10^, or one month after receiving booster doses of the respective BNT162b2 or CoronaVac or those who received two doses of CoronaVac and received a booster dose of BNT162b2 vaccine^11^. COVID-19 recovered individuals receiving one dose of vaccines were also studied. Methods used for sampling of participants, serological tests and statistical analyses are detailed in Supplementary Information.

**Figure 1:**
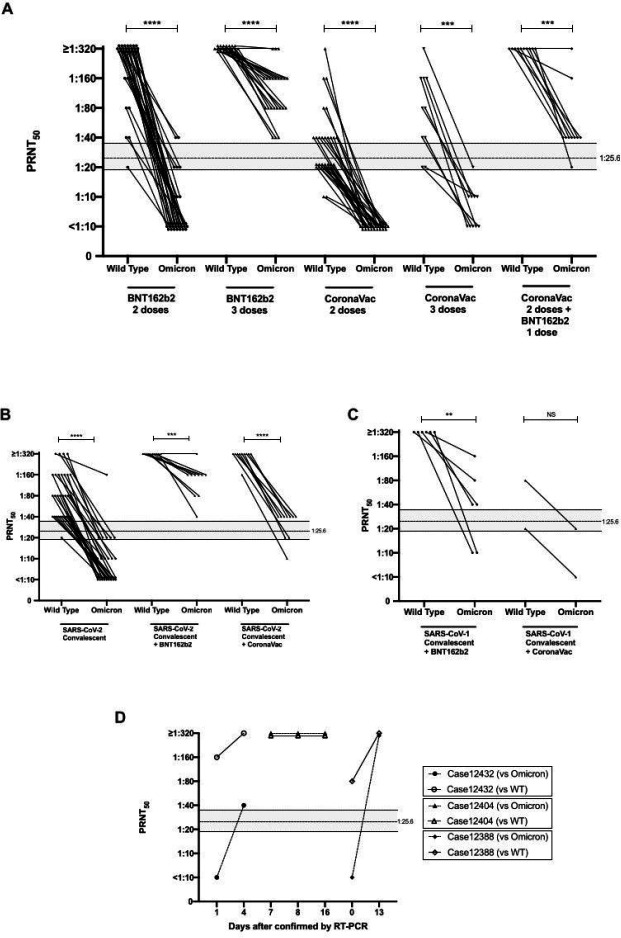
50% plaque reduction neutralization test (PRNT_50_) antibody titres (GMT and SD) to wild-type virus and Omicron variant. Mann-Whitney test was used for significance. See Table for numbers in each group. A. Individuals with 2 or 3 doses of BNT162b2 or CoronVac vaccines, as indicated. B. SARS-CoV-2 convalescent individuals with or without BNT162b2 or CoronaVac vaccine (one dose). C. SARS-1 convalescent individuals with BNT162b2 or CoronaVac vaccines. D. PRNT50 antibody titres to wild-type and Omicron variant SARS-CoV-2 viruses in three patients with SARS-CoV-2 Omicron variant infection (see supplementary information for vaccine history and clinical details). ****p<0.0001; ***p p<0.001; **p<0.01; NS p>0.05. Dotted line indicates threshold of protection and shading indicates 95% confidence intervals (see text).

The age, sex numbers of individuals studied and geometric mean PRNT_50_ titres of the different cohorts are summarized in table 1. The PRNT_50_ titres with the wild-type SARS-CoV-2 or Omicron variant in these cohorts are shown in Figure 1 and Supplementary Figure 1. PRNT_90_ data is shown in Supplementary figure 2. PRNT_50_ titres in COVID-19 vaccinated or convalescent cohorts were markedly lower to Omicron variant than to wild-type virus (Figure 1A–C, Table 1). The fold reduction in comparative GMT to Omicron vs wild-type was 30.9-fold for those receiving two doses of BNT162b2 vaccine, 10.5-fold in COVID-19 convalescent sera and 6.4-fold in those receiving two doses of CoronaVac vaccine (Figure 1A, Table 1). However, comparison of fold-difference in GMT to the two viruses is confounded by groups with low GMT to the wild-type virus because a smaller fold-reduction would already reach the detection threshold. Others report comparable reductions in neutralizing titres to Omicron variant neutralizing titres in sera of vaccinated or COVID-19 convalescent individuals^13,14^.

Using methods previously described by Khoury and colleagues^5^, we had previously determined that the threshold for 50% protection from symptomatic SARS-CoV-2 infection was a PRNT_50_ titre of 25.6 with 95% confidence intervals of 1:18.3–1:36.0^12^. Using this threshold for protection, we observed that 29 of 30 BNT162b2 vaccinated individuals retained protective PRNT_50_ antibody titres to wild-type virus but only 1 (3%) in 30 met this threshold with Omicron variant at one month after second dose of vaccine. None of those vaccinated with CoronaVac met this threshold of protective antibody with the Omicron variant (Table 1). A booster dose of BNT162b2 restored protective thresholds of Omicron variant PRNT_50_ antibody in all 23 vaccinees but a booster dose of CoronaVac in CoronaVac vaccinees did not provide protective levels of antibody. However, 9 (90%) of 10 of those receiving two doses of CoronaVac boosted with BNT162b2 had protective levels of PRNT_50_ antibody with the Omicron variant. In those who had past COVID-19 infection followed by BNT162b2 or CoronaVac vaccine, 10 of 10 and 8 of 10 respectively, had protective levels of PRNT_50_ antibody with the Omicron variant (Table 1).

It was estimated that neutralizing antibody fell by 4.7-fold within 5 to 5.8 months after the second dose of BNT162b2 vaccine and fell 6.4-fold within 6 months following CoronaVac vaccination^15,16^. Allowing for a 4.7-fold decline of PRNT_50_ antibody levels to Omicron variant, we estimated that none of the BNT162b2 or CoronaVac vaccinated individuals would retain neutralizing antibody levels above the protective threshold at 5–6 months post immunization, while 12 of 23 of those vaccinated and boosted with BTN162b2 would do so, as would 2 of 10 CoronaVac vaccinated individuals boosted with BNT162b2. None of 10 of CoronaVac vaccinated CoronaVac boosted with CoronaVac would retain PRNT_50_ antibody levels above this threshold (Table 1).

Our COVID-19 convalescent cohort was already 4.8 to 6.5 months after onset of illness and only one of 30 individuals met the protective threshold for Omicron variant. Vaccinating COVID-19 convalescent individuals with one dose of BNT162b2 or CoronaVac vaccine boosted PRNT_50_ antibody levels to above the protective threshold against Omicron. However, the levels achieved in CoronaVac vaccinated individuals was lower; and allowing for 4.7-fold waning of antibody, their PRNT_50_ would have dropped below the protective threshold although 7 of 10 who had BNT162b2 vaccinated individuals would still have protective levels of antibody to Omicron (Table 1).

We have previously shown SARS-CoV-1 convalescent sera obtained from those infected during the 2003 SARS outbreak had no neutralizing antibody to SARS-CoV-2^17^. When SARS-survivors from 2003 were vaccinated with two doses of BNT162b2, two of six elicited protective levels of PRNT_50_ antibody to Omicron but vaccination with CoronaVac (n=2) did not (Table 1).

Three returning travelers diagnosed with Omicron variant were studied as controls. They were males (range in age from 36–62) vaccinated with two doses of RNA (BNT162b2 or mRNA-1273) vaccines (see Supplementary information for details). Cases 12388 and 12432 had detectable PRNT_50_ antibody (titres of 80 and 160, respectively) to wild-type virus on the day of first RT-PCR detection or the day after, but did not have detectable cross-neutralizing antibody to Omicron variant (Figure 1D), explaining why they had breakthrough infections. They rapidly mounted neutralizing antibody to Omicron variant of ≥1:320 by day 4 (Case 12432) or day 13 (sera between day 1 and day 13 not being available for case 12388). Serum from Case 12404 was first available at days 7 by which time he had PRNT_50_ titres of ≥1:320 to both wild-type and Omicron variant. This rapid neutralizing antibody response in RNA vaccinated individuals to an antigenically divergent variant suggests that such individuals are likely to be protected from severe disease.

We included the WHO standard NIBSC 20/136 in two PRNT esperiments with PRNT_50_ titres of 1:20 and 1:40 respectively with Omicron and 1:320 with wild-type virus.

This study had some limitations. There were relatively small numbers in the individual groups, but as estimated from our power calculations (see Supplementary information), the sample size was adequate to demonstrate significant conclusions. We have not investigated other antibody activities such as binding to spike protein or binding to Fc receptors which may confer antibody-dependent cell cytotoxicity via natural killer cell mediated (ADCC). In a previous study with this cohort, we showed that both spike binding antibody and FcgRIIIa binding antibodies paralleled the PRNT antibody responses with these two vaccines^10^. However, it is possible that these antibodies may be less affected by the mutations found in the Omicron variant and may play relatively more important protective roles in protection from Omicron variant. We have not investigated T cell responses elicited by these vaccines that may provide protection, at least against severe disease.

In summary, we demonstrate that two doses of BNT162b2 or CoronaVac vaccines provide little neutralizing antibody immunity against the Omicron variant, even at one-month post vaccination. Booster doses with BNT162b2 in those with two doses of either BNT162b2 or CoronaVac provide acceptable neutralizing immunity against Omicron variant at 1-month post-booster dose. However, because three doses of BNT162b2 elicit higher levels of PRNT_50_ antibody to Omicron variant than two doses of CoronaVac followed by a booster with BNT162b2, waning antibody is likely to result in faster loss of protection with the latter schedule. It must be noted however, that CoronaVac, a whole virus inactivated vaccine with multiple viral proteins, has previously been shown to elicit a wider range to T cell responses which may compensate for some loss of neutralizing antibody protection^10^. Vaccine-efficacy studies against Omicron variant in countries where these different vaccines are being used, are urgently needed.

## Supplementary Material

Supplement 1

Supplement 2

Supplement 3

## Figures and Tables

**Table:** Age, sex and geometric mean 50% plaque reduction neutralization test (PRNT_50_) titres vs. Omicron variant

Exposure group	n	Mean Age (SD)	Age range	Male: female	WT GMT	Omicron GMT	WT; Omicron Fold GMT reduction	Above PRNT_50_ threshold of protection (1:25.6) 1 month post-vaccine		Above PRNT_50_ threshold of protection at 5–6 month post-vaccine adjusting for 4.7-fold waning	
								Omicron	Wild-type	Omicron	Wild type
BNT162b2 (2 doses)	30	51 (14.2)	25–76	16:14	216.1	7.0	30.9	1/30	29/30	0/30	25/30
BN162b2 (3 doses)	23	54.6 (14.0)	30–80	11:12	320	114.9	2.8	23/23	23/23	12/23	23/23
CoronaVac (2 doses)	30	52.1 (8.2)	39–73	10:20	32.5	5.0	6.4	0/30	14/30	0/30	3/30
CoronaVac (2 dose+CoronaVac booster)	10	48.7 (7.4)	39–59	4:6	74.6	7.6	9.7	0/10	8/10	0/10	3/10
CoronaVac (2 dose+BNT162b2 booster)	10	48.1 (7.9)	39–61	3:7	320	52.8	6.1	9/10	10/10	2/10	10/10
SARS-CoV-2 Convalescent	30	48.9 (15.8)	20–72	13:17	85.7	8.1	10.5	1/30	29/30	Not applicable	Not applicable
SARS-CoV-2 convalescent + BNT162b2 (1 dose)	10	41.6 (12.4)	26–58	5:5	320	130	2.5	10/10	10/10	7/10	10/10
SARS-CoV-2 convalescent + CoronaVac (1 dose)	10	49.9 (9.2)	37–64	5:5	298.6	30.3	9.9	8/10	10/10	0/10	10/10
SARS-1 convalescent+BNT162b2 (2 doses)*	6	48.8 (2.9)	44–52	4:2	320	35.7	9.0	4/6	6/6	1/6	6/6
SARS-1+CoronaVac (2 doses)	2	52.5 (3.6)	50–53	2:0	40	10	4	0/2	1/2	0/2	0/2
COVID-19 (Omicron) Convalescent	3	45 (14.3)	36–62	3:0	320	320	1	3/3	3/3	3/3	3/3

* One of this group received three doses of BNT162b2

## References

[1] Viana R, 2021. https://krisp.org.za/manuscripts/ZHTOWa-MEDRXIV-2021-268028v1-deOliveira.pdf

[2] World Health Organization. 2021. https://www.who.int/news/item/26-11-2021-classification-of-omicron-(b.1.1.529)-sars-cov-2-variant-of-concern

[3] Addetia A, J Clin Microbiol. 58(11):e02107–20 (2020). 32826322 10.1128/JCM.02107-20 PMC7587101

[4] McMahan K, Nature. 590:630–634 (2021). 33276369 10.1038/s41586-020-03041-6 PMC7906955

[5] Khoury DS, Nat Med. 27:1205–1211 (2021). 34002089 10.1038/s41591-021-01377-8

[6] Cromer D, Lancet Microbe. (2021) doi: 10.1016/S2666-5247(21)00267-6.

[7] Zhang Y, Lancet Infect Dis. 21:181–192 (2021). 33217362 10.1016/S1473-3099(20)30843-4 PMC7832443

[8] Jara A, N Engl J Med. In press.

[9] Bangkok Post, 2021 (https://www.bangkokpost.com/world/2133987/hundreds-of-vaccinated-indonesian-health-workers-infected).

[10] Mok CKP, Respirology Epub ahead of print.

[11] Mok CKP, Am J Resp and Crit Care Med -in press.

[12] Lau EH, EClinicalMedicine. 41:101174 (2021). 34746725 10.1016/j.eclinm.2021.101174 PMC8556690

[13] Cele S, medRxiv [Preprint]. 2021 doi: 10.1101/2021.12.08.21267417.

[14] Lu L, Clin Infect Dis (2021) Epub ahead of print.

[15] Levin EG, N Engl J Med. 385:e84. 2021. 34614326 10.1056/NEJMoa2114583 PMC8522797

[16] Zeng G, Lancet Infect Dis. 7:S1473-3099(21)00681-2. (2021).

[17] Lv H , Cell Reports 31; 107725 (2020) 10.1016/j.celrep.2020.107725 PMC7231734 32426212

