# Supplementary figures and images for: Neutralizing antibody titres to SARS-CoV-2 Omicron variant and wild-type virus in those with past infection or vaccinated or boosted with mRNA BNT162b2 or inactivated CoronaVac vaccines
Source: Res Sq. 2022 Jan 5:rs.3.rs-1207071. Preprint. [Version 1] doi: 10.21203/rs.3.rs-1207071/v1 (PMC8750710; doi:10.21203/rs.3.rs-1207071/v1)

A

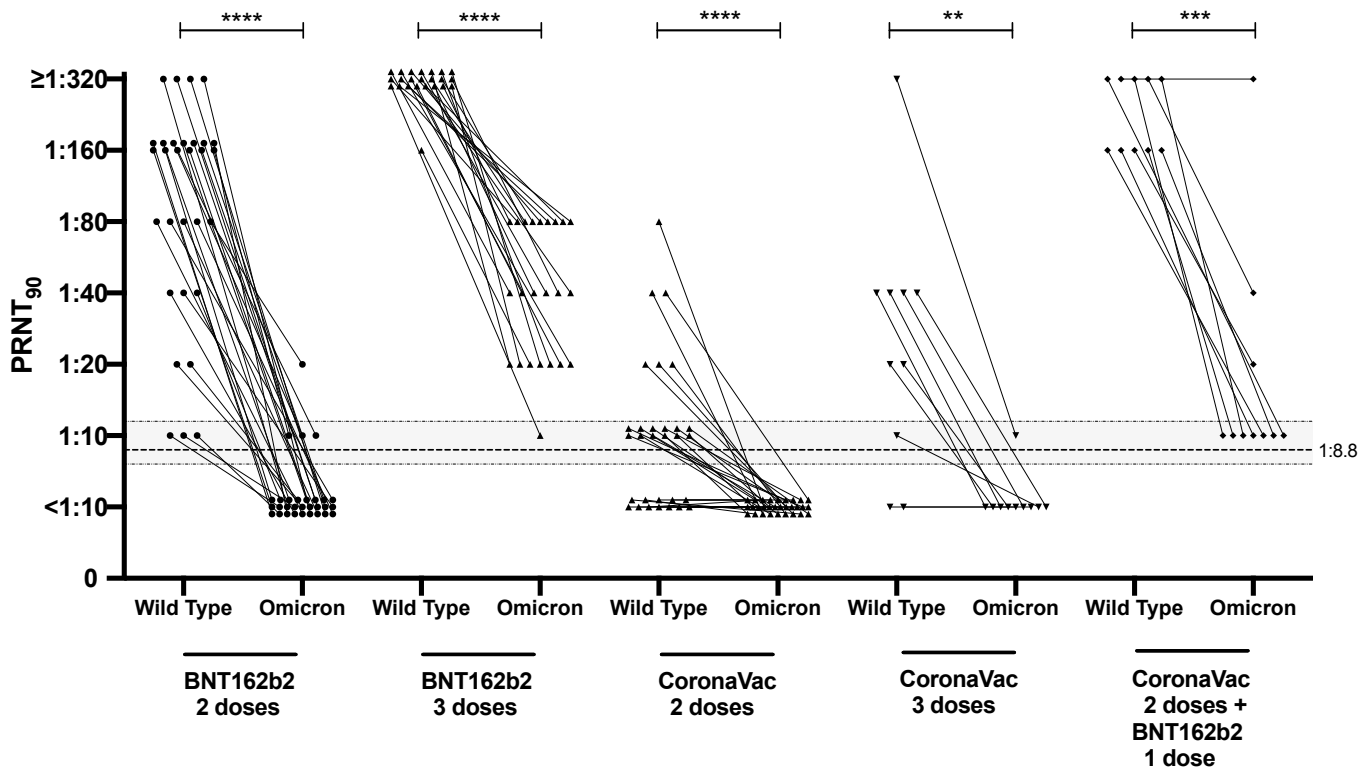

B

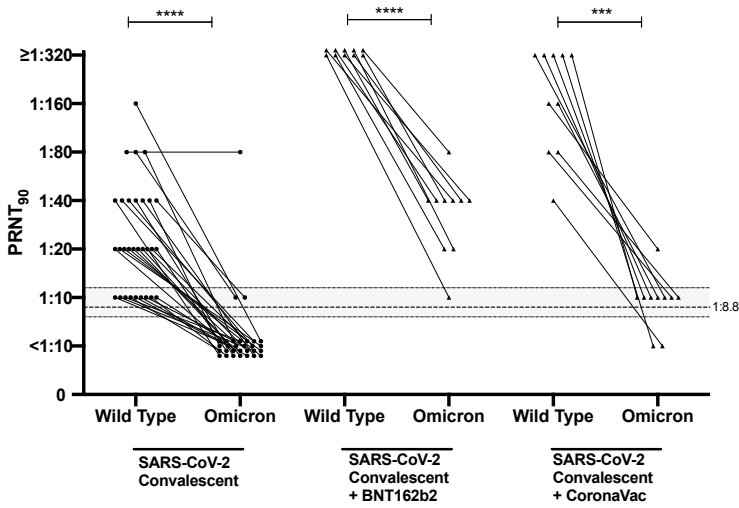

C

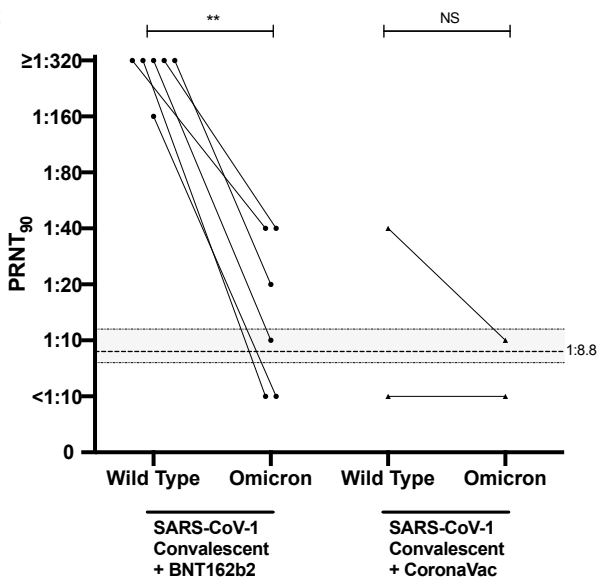

D

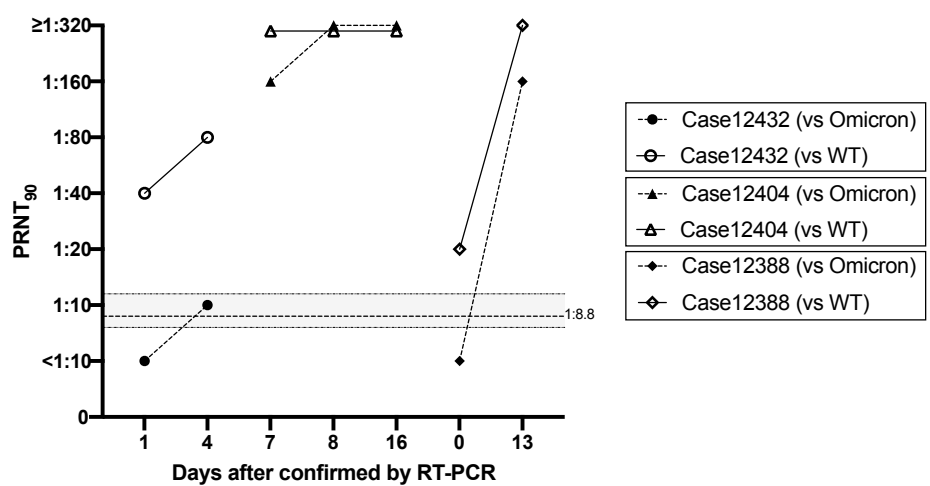

Supplement: Supplement 1 [file 284f6cd7c6b7055c3a1abe45.pdf]

A

Wild Type

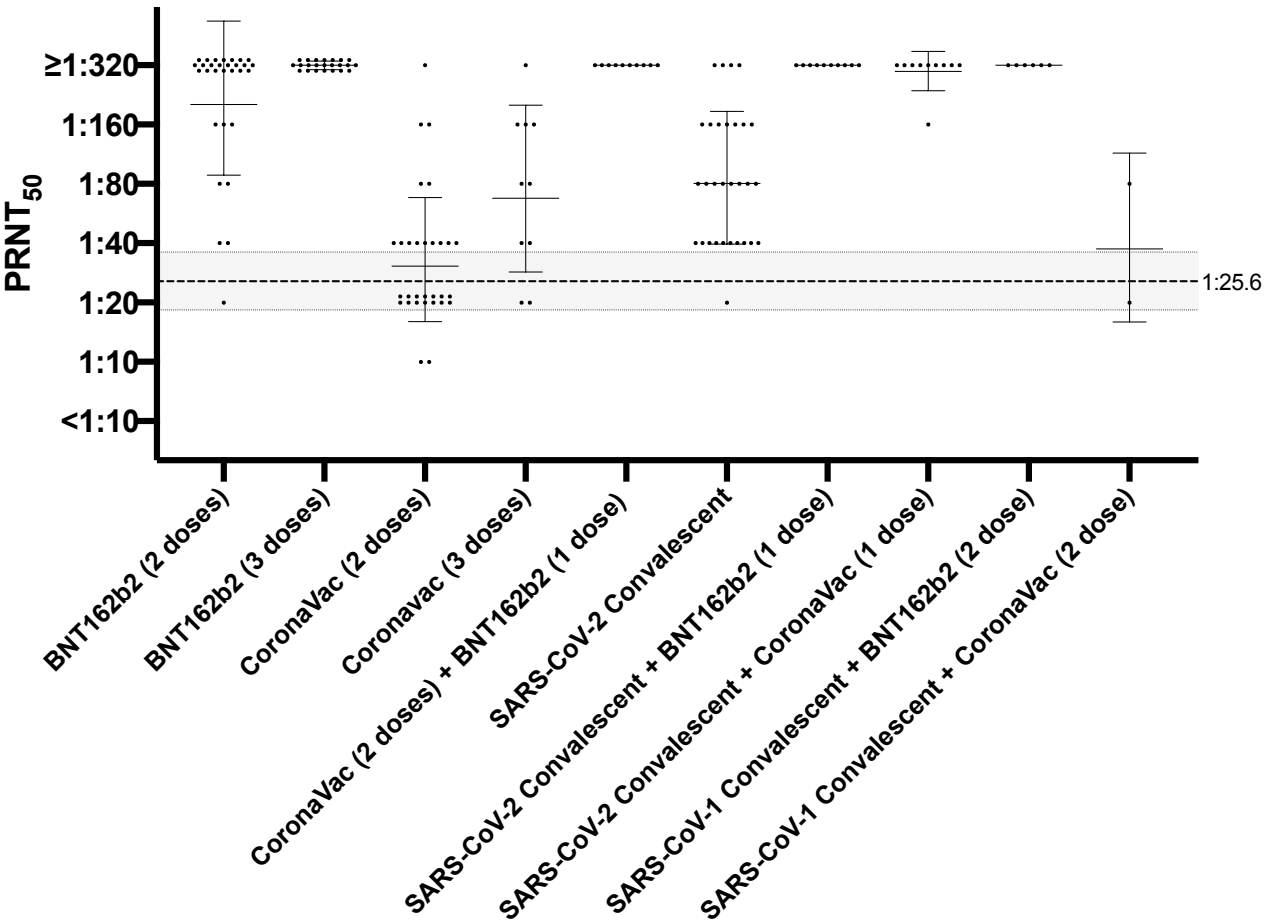

B

Omicron

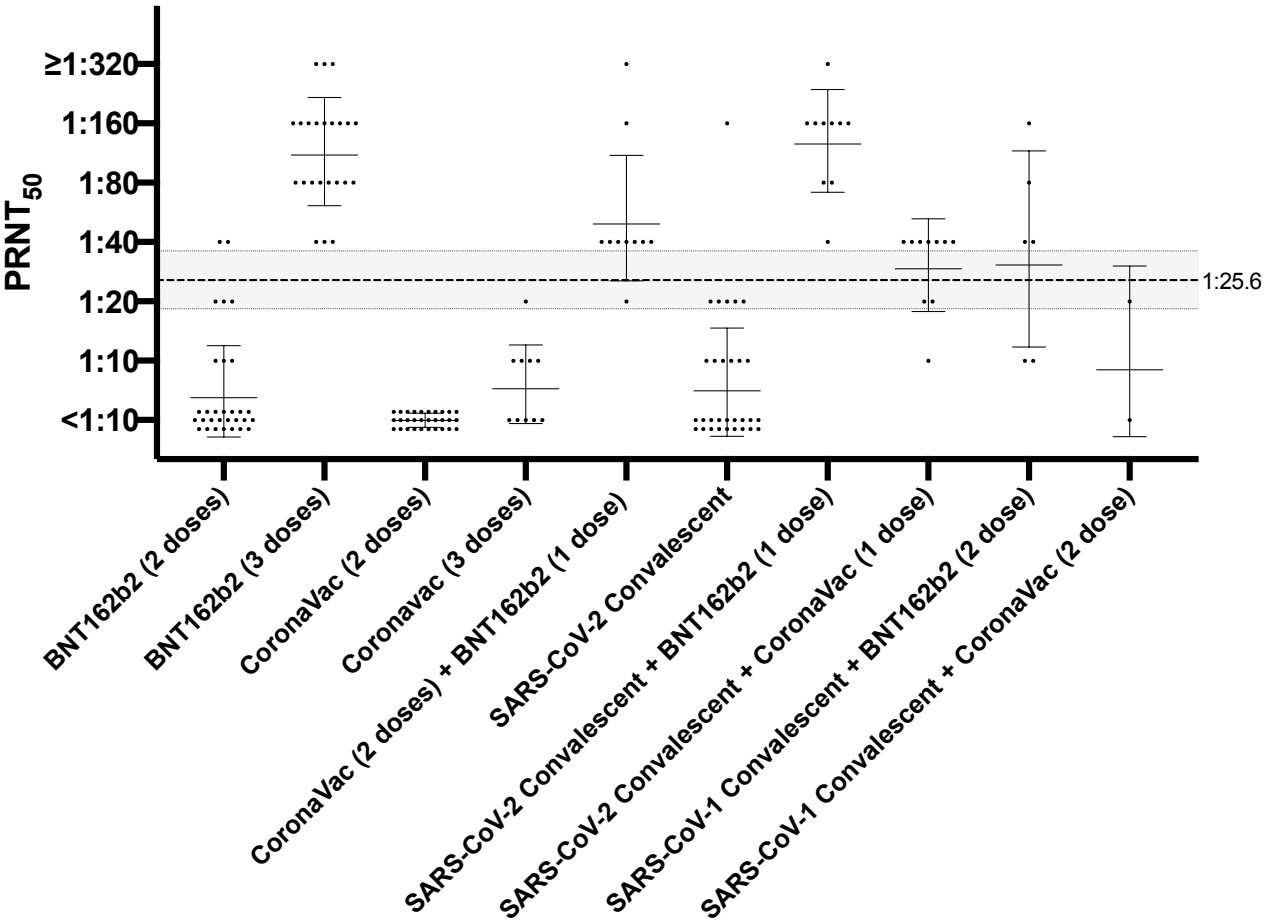

Supplement: Supplement 3 [file 9b75af0b151f01e2a12aacb6.pdf]
