# Supplementary material for: Neutralizing antibody titres to SARS-CoV-2 Omicron variant and wild-type virus in those with past infection or vaccinated or boosted with mRNA BNT162b2 or inactivated CoronaVac vaccines
Source: Res Sq. 2022 Jan 5:rs.3.rs-1207071. Preprint. [Version 1] doi: 10.21203/rs.3.rs-1207071/v1 (PMC8750710; doi:10.21203/rs.3.rs-1207071/v1)
Supplement: Supplement 2 [file 7c825ebe28c15b11858dec64.docx]

**Supplementary data**

**Methods:**

Clinical specimens: Post vaccine sera were randomly selected from cohorts of individuals included in previous studies of COVID-19 convalescent individuals^1^. Sera from day 143-196 days (4.7-6.5 months) post-infection were selected to represent waning antibody titres in convalescence. Sera collected one-month post vaccination with two doses or boosters as denoted were randomly selected from cohorts described in previous COVID-19 vaccine studies (ClinicalTrials.gov with identifier NCT04611243) ^2,3^. Individuals who had a past history of COVID-19 and had received either BNT162b2 or CoronaVac vaccine were also included and analyzed separately. SARS-CoV-1 survivors from 2003 who received either vaccine were included in the study. Sequential sera from three recent patients infected with Omicron variant infection were included as positive controls for comparison. The study was carried out in Hong Kong during the period 21^st^ February 2020 to November 20^th^ 2021. The study was approved by the Joint Chinese University of Hong Kong-New Territories East Cluster Clinical Research Ethics Committee (Ref no: 2020.229) and all participants provided informed written consent. The clinical trial was registered at ClinicalTrials.gov with identifier NCT04611243. Sample sizes for each group are indicated in Table. See statistical methods for the basis for sample size calculations.

Virology: We used a strain of Omicron variant isolated from the nose & throat swab of a returning traveler diagnosed with Pango lineage B.1.1.529 Omicron variant infection on November 13, 2021, while in hotel quarantine in Hong Kong^4^. The clinical and epidemiological details of this patient has been previously reported^4^. The virus sequence reported (GISAID Accession nos. EPI_ISL_6716902). The virus was isolated in Vero E6 cells overexpressing TMPRSS-2^5^ (kindly provided by Dr S Matsuyama and colleagues) and the passage level 3 virus aliquots were used in these studies. The cells used in the study were regularly tested for mycoplasma contamination at the Core Facility, Centre for PanorOmic Sciences, The University of Hong Kong. This virus stock used for the study was genetically sequenced and shown to have identical amino acid sequence to that found in the original clinical swab. The virus sequence from the original swab and the isolate had amino acid R346 in the spike protein, as does the majority of Omicron variant viruses to date. The Wuhan-like SARS-CoV-2 BetaCoV/Hong Kong/VM20001061/2020 (WT) isolated in Hong Kong in February 2020 was used for comparison. Culture virus stock had no amino acid changes compared to sequence from the original swab.

Plaque reduction neutralization tests were carried out on sera diluted serially from 1:10 to 1:320 using methods previously described using Vero E6 TMRESS cells^1,6^. The highest serum dilution neutralizing ≥50% and ≥90% of input plaques was regarded as the 50% plaque reduction neutralization (PRNT50) and PRNT90 titres, respectively. A virus back titration and a positive control serum was included in every experiment. The WHO NIBCS control serum NIBSC 20/136 was also included in two titrations.

Statistical analysis.

Sample size calculations: The maximum standard deviation (SD) of log titers for the uninfected vaccinated groups were 1.37 and 1.77 for PRNT50 and PRNT90 respectively. Assuming a 3-fold difference in GMT, a sample size of 10 in each group would have statistical power of >0.99 and 0.94 for PRNT_50_ and PRNT_90_ respectively, for detecting a difference between groups using Mann-Whatney U test. Comparisons between groups with larger sample size or smaller within-group variation would have larger statistical power.

Continuous variables were summarized as geometric mean with SD while categorical variables summarized as proportions or percentage. Comparison between antibody titres to wild type and Omicron variant viruses was done using the Mann-Whitney U test. P values < 0.05 were considered statistically significant.

Clinical details on three patients with Omicron disease (Figure 1D). PRNT50 antibody responses to wild type or Omicron variant SARS-CoV-2 is shown in main text figure D: Case 12388 was 36-year-old male vaccinated with two doses of BNT162b1 with second dose on 4 June 2021. Omicron variant detected by RT-PCR on 14 Nov 2021. He was asymptomatic at diagnosis and subsequent symptom was sore throat. Case 12404 was a 62-year-old male vaccinated with two doses of BNT162b2 with second dose on 25 May 2021. Omicron variant detected on 26^th^ Nov 2021. He was asymptomatic at diagnosis and subsequently developed sore throat and stuffy nose. Case 12432 was a 37-year-old male who received two doses of mRNA-1273 RNA vaccine with 2^nd^ dose on 25^th^ October 2021. Omicron variant was detected on 20 Nov 2021. He was asymptomatic at diagnosis and developed cough subsequently.

**Data availability**

The source data (individual anonymized patient and linked laboratory data) are provided

as a Source Data file available on line. Source data are provided with this paper**.** The viruses used can be obtained on request.

**References:**

1. Lau EH, et al. EClinicalMedicine. 41:101174 (2021).
2. Mok CKP, et al. Respirology Epub ahead of print.
3. Mok CKP, Am J Resp and Crit Care Med -in press.
4. Gu H et al. Emerg Infect Dis.28(2). doi: 10.3201/eid2802.212422. (2021).
5. Matsuyama S, et al. Proc Natl Acad Sci U S A. 117:7001-7003 (2020).
6. Perera RA et al. Euro Surveill. 25(16):2000421 (2020).
7. Khoury DS, et al. Nat Med. 27:1205-1211 (2021).

**Supplementary figures:**

Supplementary figure 1. Comparison of PRNT^50^ antibody titres to Wild-type (A) or Omicron Variant (B) in different groups with mean and SD. Numbers in each group indicated in table. Dotted line indicates the threshold of protection derived from method of Khoury et al^7^ and the shading indicates 95% confidence intervals of this threshold of protection.

Supplementary figure 2. 90% plaque reduction neutralization test (PRNT_90_) antibody titres to wild-type virus and Omicron variant in A. groups of individuals with 2 or 3 doses of vaccines, BNT162b2 or CoronVac as indicated. B. SARS-CoV-2 convalescent individuals with or without BNT162b2 or CoronaVac vaccine (one dose), C. SARS-1 convalescent individuals with BNT162b2 or CoronaVac vaccines. Numbers in each group indicated in table. ****p<0.0001; ***p p<0.001; **p<0.01; NS p>0.05. Dotted line indicates threshold of protection and shading indicates 95% confidence intervals (see text).
